# COVID-19-related anxiety trajectories in children, young people and adults with rheumatic diseases

**DOI:** 10.1093/rap/rkad007

**Published:** 2023-01-11

**Authors:** Stephanie J W Shoop-Worrall, Suzanne M M Verstappen, Wendy Costello, Saskya P Angevare, Yosef Uziel, Carine Wouters, Nico Wulffraat, Richard Beesley

**Affiliations:** Centre for Health Informatics, University of Manchester, Manchester, UK; Centre for Epidemiology Versus Arthritis, University of Manchester, Manchester, UK; Centre for Epidemiology Versus Arthritis, University of Manchester, Manchester, UK; NIHR Manchester BRC, Manchester University NHS Foundation Trust, Manchester Academic Health Science Centre, Manchester, UK; European Network for Children with Arthritis, Geneva, Switzerland; iCAN Ireland, Bansha, Ireland; European Network for Children with Arthritis, Geneva, Switzerland; KAISZ, Amsterdam, The Netherlands; Pediatric Rheumatology Unit, Meir Medical Center, Kfar Saba, Israel; Department of Pediatrics, Sackler School of Medicine, Tel Aviv, Israel; Pediatric Rheumatology Division, University Hospitals Leuven, Leuven, Belgium; Department of Microbiology and Immunology, KU Leuven University, Leuven, Belgium; Department of Pediatric Rheumatology, Wilhelmina Children's Hospital, UMC Utrecht, Utrecht, The Netherlands; European Network for Children with Arthritis, Geneva, Switzerland; Juvenile Arthritis Research, Tonbridge, UK

**Keywords:** COVID-19, rheumatology, anxiety

## Abstract

**Objectives:**

Uncertainty regarding the risk of coronavirus disease 2019 (COVID-19), its complications and the safety of immunosuppressive therapies may drive anxiety among adults and parents of children and young people (CYP) with rheumatic diseases. This study explored trajectories of COVID-related anxiety in adults and parents of CYP with rheumatic diseases.

**Methods:**

Adults and parents of CYP participating in the international COVID-19 European Patient Registry were included in the current study if they had enrolled in the 4 weeks following 24 March 2020. COVID-related anxiety scores (0–10) were collected weekly for up to 28 weeks.

Group-based trajectory models explored COVID-related anxiety clusters in adult and parent populations, with optimal models chosen based on model fit, parsimony and clinical plausibility. Demographic, clinical and COVID-19 mitigation behaviours were compared between identified clusters using univariable statistics.

**Results:**

In 498 parents of CYP and 2640 adults, four common trajectory groups of COVID-related anxiety were identified in each cohort: persistent extreme anxiety (32% and 17%), persistent high anxiety (43% and 41%), improving high anxiety (25% and 32%) and improving moderate anxiety (11% and 10%), respectively. Few characteristics distinguished the clusters in the parent cohort. Higher and more persistent anxiety clusters in the adult cohort were associated with higher levels of respiratory comorbidities, use of immunosuppressive therapies, older age and greater self-isolation.

**Conclusions:**

COVID-19-related anxiety in the rheumatic disease community was high and persistent during the COVID-19 pandemic, with four common patterns identified. In the adult cohort, higher COVID-related anxiety was related to perceived risk factors for COVID-19 morbidity and mortality.

Key messages

- Coronavirus disease 2019 (COVID-19)-related anxiety is persistently high in adults and parents of CYP with rheumatic diseases.
- Higher and more persistent anxiety in adults with rheumatic diseases is related to perceived COVID-19 risk factors.
- Immunosuppressant safety during the COVID-19 pandemic should be shared with the rheumatic disease community.

## Introduction

The coronavirus disease 2019 (COVID-19) pandemic has prompted dramatic changes to everyday lifestyles. Social distancing, self-isolation and limited time spent engaging in social, recreational and physical activities have become the new normal [1, 2]. As children and young people (CYP) are kept in their homes and away from schools, colleges and universities, parents and caregivers have been forced to home school and/or supervise online learning alongside their own occupations [3], with many facing long-term furlough, unemployment or temporary career changes [4]. While such lifestyle changes reduce the overall spread of COVID-19 [5], they also place a high burden on the mental health of both young and older people [6, 7].

Children, young people and adults with rheumatological conditions may be at increased risk of contracting or experiencing greater complications from COVID-19 due to a combination of autoimmune conditions and commonly prescribed immunosuppressive therapies [8, 9]. There has been great uncertainty regarding whether this group of people should be self-isolating over prolonged periods or participating in face-to-face teaching, work and/or other social activities. The potential increased risk due to autoimmune disease, coupled with uncertainty regarding medication safety and safe pandemic lifestyles, may place additional mental health burdens on people with rheumatic diseases and their families, who may be anxious about their own and their family’s safety.

Unsupervised machine learning methods, such as latent class trajectory modelling, can identify different patterns in outcomes, such as anxiety, that may differ between groups, or clusters, of people [10]. It is currently unknown whether people with rheumatic diseases view their risk of contracting COVID-19 similarly and have similar levels of anxiety or whether distinct clusters may experience different patterns of anxiety that may have changed since the initial outbreak. Identifying these clusters and distinguishing characteristics of people between clusters aids in understanding the mental health burden on people with rheumatic disease and who may be in need of greater support during the COVID-19 pandemic.

This study explored the burden of COVID-related anxiety and whether there are clusters of people with different patterns of anxiety among adults and parents of children and young people with rheumatic diseases recruited globally since March 2020.

## Methods

### Study population

The COVID-19 European Patient Registry is an international, parent-led, online, self-referred prospective cohort. It was initiated in March 2020 to understand the impact of the COVID-19 pandemic on people with rheumatic diseases. As such, it recruits people of all ages from around the globe, specifically targeting those with rheumatic diseases, but it is also open to those with no rheumatic disease. Since this was a parent-/patient-led study comprising online, self-referred and completed questionnaires, healthcare ethical approval was not required, as confirmed by the National Health Service Research Ethics Committees online tools (http://www.hra-decisiontools.org.uk/ethics/). However, written informed consent was provided by adults and parents/guardians of CYP with rheumatic diseases. For the current study, participants with a rheumatic disease were selected if they enrolled in the registry during the 4 weeks following 24 March 2020, to allow for at least 6 months of follow-up.

### Data collection

Two sets of questions were provided as part of the online survey: a set targeted towards CYP, for completion by a parent or caregiver, and a set targeted towards adults for self-completion. Questionnaires were provided in 13 languages. At initial enrolment, all participants were asked for information about their demographics (gender, age, country of residence), rheumatic disease (specific diagnoses, current disease burden, medications, comorbidities) and factors related to COVID-19, including mitigation methods (isolation, social distancing, none). Following enrolment, weekly surveys ascertained any changes in medication, rheumatic disease control, contact with healthcare professionals and COVID factors.

### Outcome

As part of the initial and follow-up questionnaires, participants were asked to rate their anxiety related to COVID-19 on a scale from 0 to 10, ranging from ‘not at all worried’ (0) to ‘extremely worried’ (10), based on the following question: ‘On a scale of 0 to 10, how worried are you about coronavirus affecting [you/your child]?’.

### Clustering and subgroup discovery

Group-based trajectory models were used to cluster participants based on shared patterns of COVID-19-related anxiety over the 6 months following enrolment. Models were built separately for CYP and adult cohorts. The models were constructed using censored normal distributions and were tested using 1–10 trajectory groups within linear, quadratic and cubic polynomial trajectory shapes [10].

Models with a cluster representing <1% of the cohort were excluded, as were those with average posterior probabilities for group membership <70% or relative entropy <0.5. From the remaining shortlist, an optimal model was selected for each CYP and adult cohort using a combination of statistical fit (Bayesian information criteria), parsimony and clinical plausibility [10]. Clusters were labelled based on subjective assessments of overall trajectory patterns.

### Characteristics of each group

Characteristics collected at enrolment (demographics, rheumatic disease, COVID mitigation behaviours) were compared between identified clusters descriptively and through univariable statistics. Differences in categorical variables were explored via chi-squared or Fisher’s exact tests and continuous variables via Kruskal–Wallis metrics.

### Missing data

No imputations of missing data were undertaken for the outcome since group-based trajectory modelling is robust to bias from missing-at-random data, as a maximum likelihood–based technique. Data were analysed using Stata 14 (StataCorp, College Station, TX, USA). Data visualization was completed in R version 3.6.1 (R Foundation for Statistical Computing, Vienna, Austria) using RStudio version 1.2.5001.

## Results

### Study population

Within the CYP (*n* = 498) and adult (*n* = 2640) cohorts, the majority were female (65% and 85%, respectively) and the most common region of residence was the UK (50% and 84%, respectively). In the CYP cohort, the most common rheumatic disease diagnoses were polyarticular JIA (37%) and oligoarticular JIA (29%), with RA most common in the adult cohort (63%). Most participants were taking at least one immunosuppressive therapy (85% and 87%, respectively) and respiratory comorbidities were uncommon (10% and 17%, respectively). At recruitment, 88% in the CYP cohort and 79% in the adult cohort were self-isolating (Supplementary Tables S1 and S2, available at *Rheumatology Advances in Practice* online).

### Trajectory clusters of anxiety

**Figure 1.**
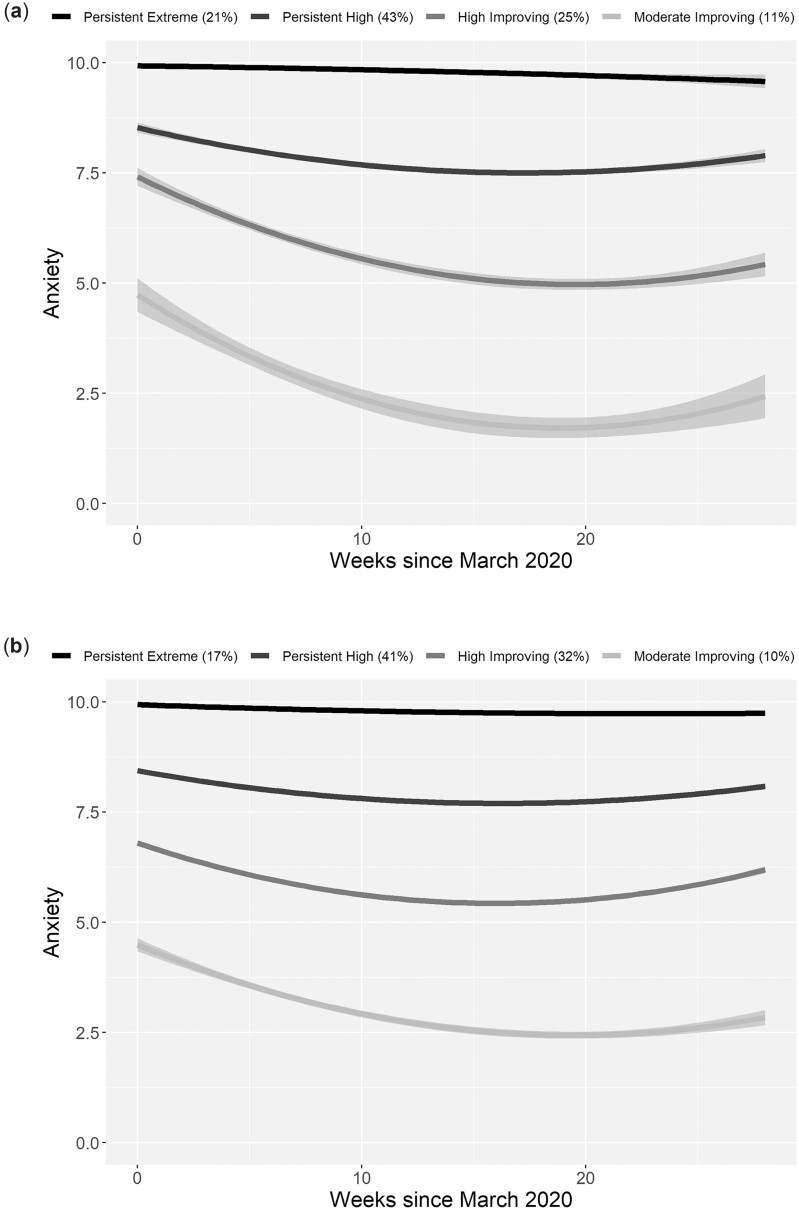
Trajectories within clusters of COVID-related anxiety in the 6 months following March 2020 in **(a)** parents of CYP and **(b)** adults with rheumatic diseases

Four trajectory clusters of COVID-19-related anxiety were identified in the CYP and adult cohorts with similar patterns: persistent extremely high anxiety (32% and 17%), persistent high anxiety (43% and 41%), high anxiety that marginally improved (25% and 32%) and moderate anxiety that improved (11% and 10%), respectively (Fig. 1, Supplementary Figs S1 and S2, available at *Rheumatology Advances in Practice* online).

### Characteristics of people within each anxiety cluster

Few features within the CYP cohort differed between the anxiety clusters. Only country of residence was significantly associated with anxiety, with greater representation of the UK, Ireland, Italy and Slovenia in the highest anxiety groups and greater representation of France, Spain and the USA in the lower anxiety groups (Supplementary Fig. S3, Supplementary Table S3, available at *Rheumatology Advances in Practice* online).

Among the adult cohort, a greater number of features distinguished the clusters. Those with greater and more persistent anxiety had higher use of immunosuppressive therapies (extreme, 87%; high, 90%; high improvers, 86%; moderate improvers, 82%), higher levels of respiratory comorbidities (extreme, 25%; high, 16%; high improvers, 14%; moderate improvers, 14%) and higher levels of initial self-isolation (extreme, 86%; high, 81%; high improvers, 75%; moderate improvers, 70%) and were marginally older (Table 1).

**Table 1:** Univariable demographic, psychosocial, COVID-19 mitigation behaviours and disease features of patients in each anxiety trajectory group in the COVID-19 European Patient Registry: adults

Characteristics at recruitment	Cluster				*P*-value
	Persistent extreme	Persistent high	High improving	Moderate improving	
*N* (%)	453 (17)	1101 (41)	856 (32)	265 (10)	–
Demographics					
Country of residence, *n* (%)					<0.001
Canada	1 (<1)	6 (1)	2 (<1)	1 (<1)	
Greece	1 (<1)	13 (1)	17 (2)	12 (5)	
Ireland	8 (2)	14 (1)	13 (2)	5 (2)	
Israel	6 (1)	17 (2)	11 (1)	4 (2)	
Italy	8 (2)	12 (1)	20 (2)	9 (3)	
Netherlands	0 0	3 (<1)	7 (1)	1 (<1)	
Spain	5 (1)	10 (1)	19 (2)	4 (2)	
Sweden	2 (<1)	5 (<1)	16 (2)	3 (1)	
USA	10 (20	25 (2)	30 (4)	6 (2)	
UK	400 (88)	964 (88)	681 (80)	203 (77)	
Other	12 (3)	32 (3)	40 (5)	17 (6)	
Age, years, median (IQR)	51 (42–59)	52 (43–60)	50 (40–59)	50 (37–61)	0.008
Gender, *n* (%)					0.147
Female	414 (91)	993 (90)	756 (88)	223 (84)	
Male	39 (9)	106 (10)	98 (11)	41 (15)	
Non-binary	0 0	1 (<1)	1 (<1)	1 (<1)	
Prefer not to say	0 0	1 (<1)	1 (<1)	0 0	
Clinical—RMD					
Diagnosis group, *n* (%)					<0.001
Autoinflammatory	6 1(0	16 (1)	18 (2)	9 (3)	
Axial SpA	11 (2)	36 (3)	32 (4)	7 (3)	
Systemic JIA	7 (2)	8 (1)	10 (1)	3 (1)	
JIA	10 (2)	34 (3)	43 (5)	10 (4)	
Psoriatic arthritis	23 (5)	70 (6)	55 (6)	11 (4)	
RA	297 (66)	733 (67)	507 (59)	147 (55)	
Still’s disease	4 (1)	9(1)	1 (<1)	2 (1)	
SLE	15 (3)	30 (3)	21 (2)	9 (3)	
RA plus others	11 (2)	24 (2)	26 (3)	10 (4)	
Other	68 (15)	136 (12)	126 (15)	45 (17)	
Control of RMD, median (IQR)	7 (5–9)	7 (5–9)	7 (5–9)	7 (5–9)	0.006
Respiratory comorbidity	114 (25)	180 (16)	121 (14)	38 (14)	<0.001
Immunosuppressive therapy	395 (87)	988 (90)	740 (86)	217 (82)	0.004
COVID-related, *n* (%)					
Ever had COVID	7 (2)	11 (1)	16 (2)	3 (1)	0.410
COVID mitigation behaviours					
Self-isolation/isolation/quarantine	379 (86)	861 (81)	621 (75)	181 (70)	<0.001
Distancing	205 (47)	564 (53)	487 (58)	156 (60)	<0.001
None	5 (1)	10 (1)	11 (1)	8 (3)	0.055

IQR, interquartile range; RMD, rheumatic and musculoskeletal diseases.

## Discussion

The current study identified four common patterns of COVID-19-related anxiety that are consistent across adults and parents of CYP with rheumatic diseases recruited globally. Anxiety levels were high and persistent among most of the cohort, with only two of four clusters experiencing any substantial improvement in anxiety in the 6 months following March 2020. Although there were few features in parents of children with rheumatic disease that distinguished the clusters, characteristics that may be associated with a higher risk of COVID-19 morbidity and mortality were associated with assignment to higher and more persistent anxiety clusters among adults with rheumatic diseases.

The four anxiety trajectories identified in both the CYP and adult cohorts in the current study represent an exceptionally high burden of anxiety in the rheumatic disease community. Approximately one-third of parents of CYP with rheumatic disease experienced extremely high anxiety (median 10/10) that persisted over the 6 months. Over 40% of both cohorts experienced high anxiety that also persisted over the 6 months with little improvement. The similarity between the CYP and adult cohorts may reflect high anxiety in the general population that has translated to these potentially vulnerable people, with estimates of anxiety symptoms in the general population of several countries ranging from 6.3 to 50.9% during the COVID-19 pandemic [11]. However, the similarities in anxiety patterns could also reflect similar worries across the rheumatic disease community regarding their own or their children’s conditions, medication and uncertainty whether they should be avoiding face-to-face occupations, schooling and social activities.

Few factors were associated with anxiety clusters in parents of CYP with rheumatic diseases. The only significant factor was country of residence, which may be associated with different COVID-19 policies, guidelines and infection and morbidity/mortality rates, in addition to overall cultural differences that may influence anxiety. Factors related to the burden of COVID-19 infection were not related to anxiety, suggesting persistent high levels of anxiety regardless of risk. Therefore there is a clear unmet need for anxiety management programs or interventions for parents of CYP with rheumatic diseases, whose anxiety is high despite their children being at low risk of infection or complications from COVID-19 [12].

In the adult cohort, factors that may be associated with COVID-19 morbidity and mortality, such as respiratory comorbidities [13], older age and use of immunosuppressive medication [9], were associated with higher and more persistent anxiety groups. These groups were also more likely to self-isolate, perhaps due to increased anxiety regarding COVID risk. Thus COVID-19 perceived risk factors associated with anxiety in the adult cohort present modifiable targets for reducing this mental health burden. To date, the use of immunosuppressive therapies has had mixed associations with the risk of contracting or experiencing poor outcomes following COVID-19 infection. Chronic moderate or high-dose corticosteroids have been associated with increased mortality and complications [9, 14], and low-dose corticosteroids and conventional and biologic DMARDs have either not been associated with COVID-19 infection or complications [9, 15] or have been associated with improved outcomes [9, 14]. Interrupting these therapies for the duration of the pandemic would likely result in an increased burden of rheumatic disease. Conversely, self-isolating while continuing immunosuppressive therapies presents a further impact on mental health. Therefore, this adult population may benefit from anxiety reduction methods that include greater distribution of information regarding immunosuppressants and COVID-19 risk.

This study benefitted from a large international cohort obtained by recruiting people across age ranges using surveys in several languages. As an online survey, the study could continue to safely recruit and gather a large volume of pandemic-specific data even during a global outbreak. In addition, responses to most questions were compulsory, which limited the amount of missing data. Finally, novel machine learning methods were implemented to identify several unique clusters of anxiety. By identifying distinct COVID-19-related anxiety patterns, the current study has identified key risk factors for high and persistent anxiety. These factors may be used to target interventions to people in specific disease clusters and/or prevent COVID-19-related anxiety across the rheumatic disease population.

The limitations of this study include the location of the study population, most of whom were based in the UK, which limited generalizability to countries where fewer participants were recruited. In addition, the outcome of anxiety was tested using a COVID-19-specific question. While this question has not been formally validated, it was considered the optimal method to ascertain levels of anxiety quickly, and specifically relating to COVID-19 risk, at the start of the pandemic. This study may have benefitted from investigating anxiety levels in CYP themselves, with the current data allowing for the evaluation of parental anxiety in this population. Similarly, it was unclear whether anxiety patterns observed were related to factors not captured by the study, such as governmental policies, key worker status, in-person *vs* online schooling and caregiver responsibilities or infection of family members or other contacts of study participants. While the study could not adjust for underlying anxiety disorders in participants, the picture of high to extreme high COVID-19-related anxiety highlights an area for mental health intervention, regardless of pre-existing diagnoses.

## Conclusion

Four common patterns of COVID-19-related anxiety were identified in adults and in parents of CYP with rheumatic diseases. For the majority of the cohort, anxiety was high and persistent in the 6 months following March 2020, during the COVID-19 pandemic. Few factors were associated with anxiety burden in parents. Factors potentially related to COVID-19 morbidity and mortality were associated with higher and more persistent anxiety in adults with rheumatic diseases. Interventions for anxiety should target the entire population of people with rheumatic disease and specifically promote information regarding the safety of immunosuppressive therapies during the COVID-19 pandemic.

## Supplementary Material

rkad007_Supplementary_Data

## Data Availability

The data underlying this article cannot be shared publicly due for the privacy of individuals who participated in the study. Please contact Richard Beesley (hello@jarproject.org) to apply for access to these data.
