# Supplementary material for: COVID-19-related anxiety trajectories in children, young people and adults with rheumatic diseases
Source: Rheumatol Adv Pract. 2023 Jan 11;7(1):rkad007. doi: 10.1093/rap/rkad007 (PMC9890081; doi:10.1093/rap/rkad007)
Supplement: rkad007_Supplementary_Data [file rkad007_supplementary_data.docx]

**SUPPLEMENTARY MATERIALS**

**Supplementary Table S1.** Baseline characteristics of the cohort: Children and Young People

| **Characteristic** | **% available** | **N (%) or Median (IQR)** |
| --- | --- | --- |
| **Demographics** | | |
| Country of residence | 100 |  |
| France |  | 65 (13) |
| Greece |  | 14 (3) |
| Ireland |  | 59 (12) |
| Italy |  | 12 (2) |
| Slovenia |  | 13 (3) |
| Spain |  | 24 (5) |
| USA |  | 13 (3) |
| UK |  | 251 (50) |
| Other |  | 50 (10) |
| Age | 100 | 11 (7, 14) |
| Female | 100 | 328 (65) |
| **Clinical - RMD** | | |
| Diagnosis group | 100 |  |
| Autoinflammatory |  | 28 (6) |
| CRMO |  | 17 (3) |
| Systemic JIA |  | 36 (7) |
| Oligoarticular JIA |  | 147 (29) |
| Polyarticular JIA |  | 186 (37) |
| Enthesitis-related JIA |  | 28 (6) |
| Psoriatic JIA |  | 31 (6) |
| Other |  | 25 (5) |
| Control of RMD | >99 | 8 (6, 10) |
| Respiratory comorbidity | 100 | 49 (10) |
| Immunosuppressive therapy | 100 | 426 (85) |
| **COVID-related** | | |
| Ever had COVID | 100 | 6 (1) |
| COVID mitigation behaviours | 476 |  |
| Self-isolation/ isolation/ quarantine |  | 421 (88) |
| Distancing |  | 238 (50) |
| None |  | 5 (1) |

**Supplementary Table S2.** Baseline characteristics of the cohort: Adults

| Characteristic | % available | N (%) or Median (IQR) |
| --- | --- | --- |
| Demographics | | |
| Country of residence | 100 |  |
| Canada |  | 10 (<1) |
| Greece |  | 43 (2) |
| Ireland |  | 40 (2) |
| Israel |  | 38 (1) |
| Italy |  | 49 (2) |
| Netherlands |  | 11 (<1) |
| Spain |  | 38 (1) |
| Sweden |  | 26 (1) |
| USA |  | 71 (3) |
| UK |  | 2248 (84) |
| Other |  | 101 (4) |
| Age (years) | 100 | 51 (41, 60) |
| Gender | 100 |  |
| Female |  | 2386 (89) |
| Male |  | 284 (11) |
| Non-binary |  | 3 (<1) |
| Prefer not to say |  | 2 (<1) |
| Clinical - RMD | | |
| Diagnosis group | 100 |  |
| Autoinflammatory |  | 49 (2) |
| Axial spondyloarthritis |  | 86 (3) |
| Systemic JIA |  | 28 (1) |
| JIA |  | 97 (4) |
| Psoriatic arthritis |  | 159 (6) |
| RA |  | 1684 (63) |
| Still’s disease |  | 16 (<1) |
| SLE |  | 75 (3) |
| RA plus others |  | 71 (3) |
| Other |  | 375 (14) |
| Control of RMD |  |  |
| Respiratory comorbidity | 100 | 453 (17) |
| Immunosuppressive therapy | 100 | 2340 (87) |
| COVID-related | | |
| Ever had COVID | 100 | 37 (1) |
| COVID mitigation behaviours | 97 |  |
| Self-isolation/ isolation/ quarantine |  | 2042 (79) |
| Distancing |  | 1412 (54) |
| None |  | 34 (1) |

**Supplementary Table S3.** Univariable demographic, psychosocial, COVID-19 mitigation behaviours and disease features of patients in each anxiety trajectory group in COVID-19 European Patient Registry: Children and young people

| **Characteristic at recruitment** | **Cluster (N, % or median, IQR)** | | | | **P-value** |
| --- | --- | --- | --- | --- | --- |
|  | **Persistent Extreme** | **Persistent High** | **High Improving** | **Moderate Improving** |  |
| N | 105 (21) | 213 (43) | 130 (25) | 53 (11) | - |
| **Demographics** | | | | | |
| Country of residence | | | | | <0.001 |
| France | 4 (3) | 27 (13) | 25 (19) | 9 (17) |  |
| Greece | 3 (3) | 6 (3) | 1 (1) | 4 (8) |  |
| Ireland | 16 (15) | 22 (10) | 19 (15) | 2 (4) |  |
| Italy | 5 (5) | 4 (2) | 2 (2) | 1 (2) |  |
| Slovenia | 6 (6) | 4 (2) | 2 (2) | 1 (2) |  |
| Spain | 1 (1) | 14 (7) | 2 (2) | 7 (13) |  |
| USA | 1 (1) | 7 (3) | 2 (1) | 3 (6) |  |
| UK | 63 (60) | 103 (49) | 67 (51) | 18 (34) |  |
| Other | 6 (6) | 26 (12) | 10 (8) | 8 (15) |  |
| Age | 10 (7, 14) | 10 (7, 13) | 11 (9, 14) | 12 (8, 14) | 0.355 |
| Female | 64 (61) | 142 (67) | 91 (70) | 31 (58) | 0.333 |
| **Clinical - RMD** | | | | | |
| Diagnosis group | | | | | 0.235 |
| Autoinflammatory | 6 (6) | 11 (5) | 6 (5) | 5 (9) |  |
| CRMO | 4 (4) | 7 (3) | 4 (3) | 2 (4) |  |
| Systemic JIA | 7 (7) | 18 (8) | 7 (6) | 4 (8) |  |
| Oligoarticular JIA | 28 (27) | 63 (30) | 39 (30) | 17 (32) |  |
| Polyarticular JIA | 33 (31) | 84 (39) | 51 (39) | 18 (34) |  |
| Enthesitis-related JIA | 4 (4) | 10 (5) | 10 (8) | 4 (8) |  |
| Psoriatic JIA | 12 (11) | 10 (5) | 9 (7) | 0 (0) |  |
| Other | 11 (10) | 7 (3) | 4 (3) | 3 (6) |  |
| Control of RMD | 8 (6, 10) | 8 (5, 10) | 8 (5, 10) | 9 (7, 10) | 0.369 |
| Respiratory comorbidity | 11 (10) | 21 (10) | 13 (10) | 4 (8) | 0.947 |
| Immunosuppressive therapy | 82 (78) | 182 (85) | 118 (91) | 44 (83) | 0.057 |
| **COVID-related** | | | | | |
| Ever had COVID | 1 (1) | 2 (1) | 2 (1) | 1 (2) | 0.916 |
| COVID mitigation behaviours |  |  |  |  |  |
| Self-isolation/ isolation/ quarantine | 90 (92) | 182 (91) | 110 (86) | 39 (80) | 0.089 |
| Distancing | 44 (45) | 100 (50) | 66 (52) | 28 (57) | 0.542 |
| None | 1 (1) | 2 (1) | 0 (0) | 2 (4) | 0.127 |

1.
2.
3.

**Supplementary Figure S1.** Percentage in each trajectory group with increasing clusters and BIC in group-based trajectory models in the CYP cohort a) linear-only, b) quadratic-only, c) cubic only.

1.
2.
3.

**Supplementary Figure S2.** Percentage in each trajectory group with increasing clusters and BIC in group-based trajectory models in the adult cohort a) linear-only, b) quadratic-only, c) cubic only.

1.
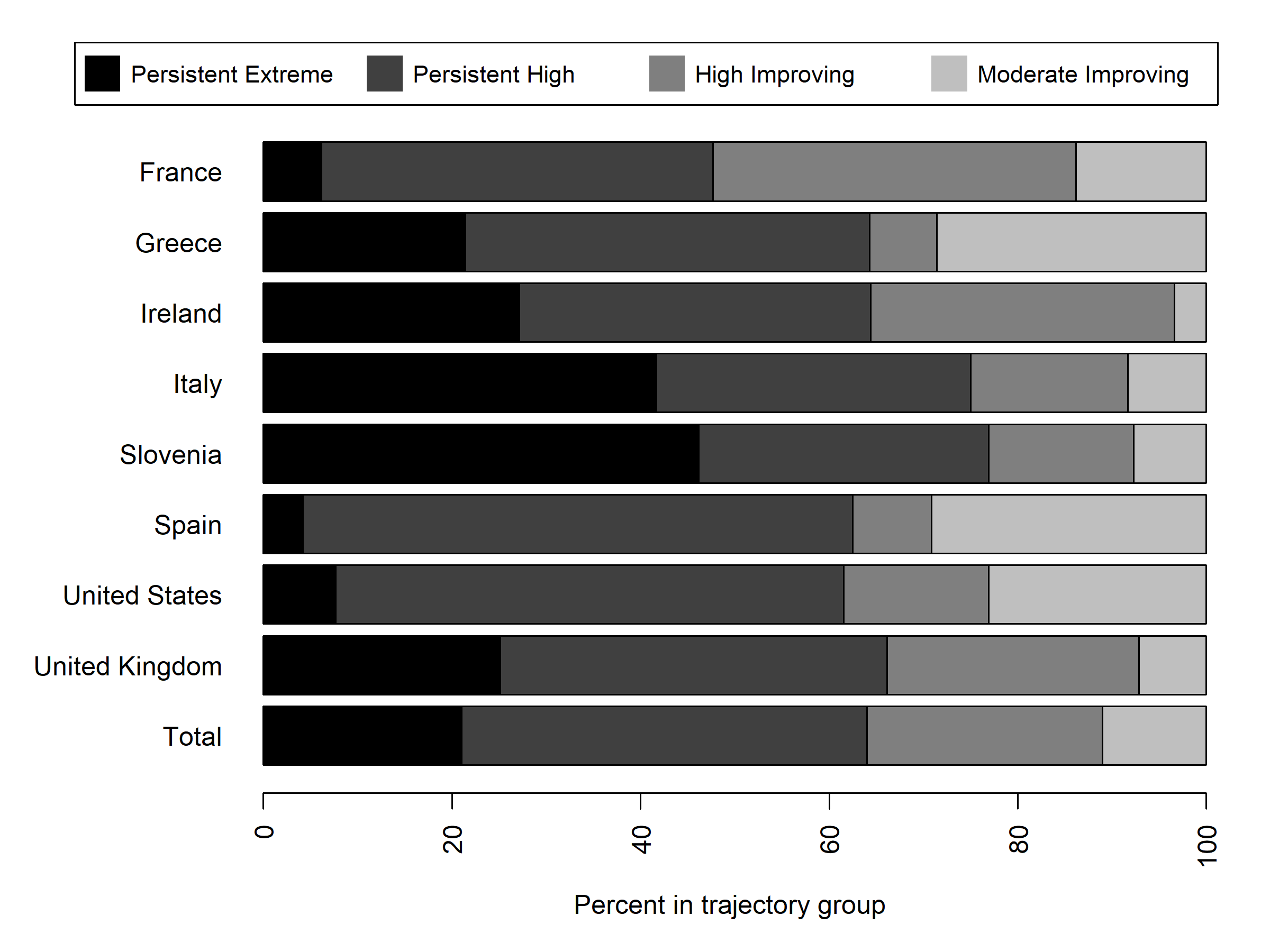

2.
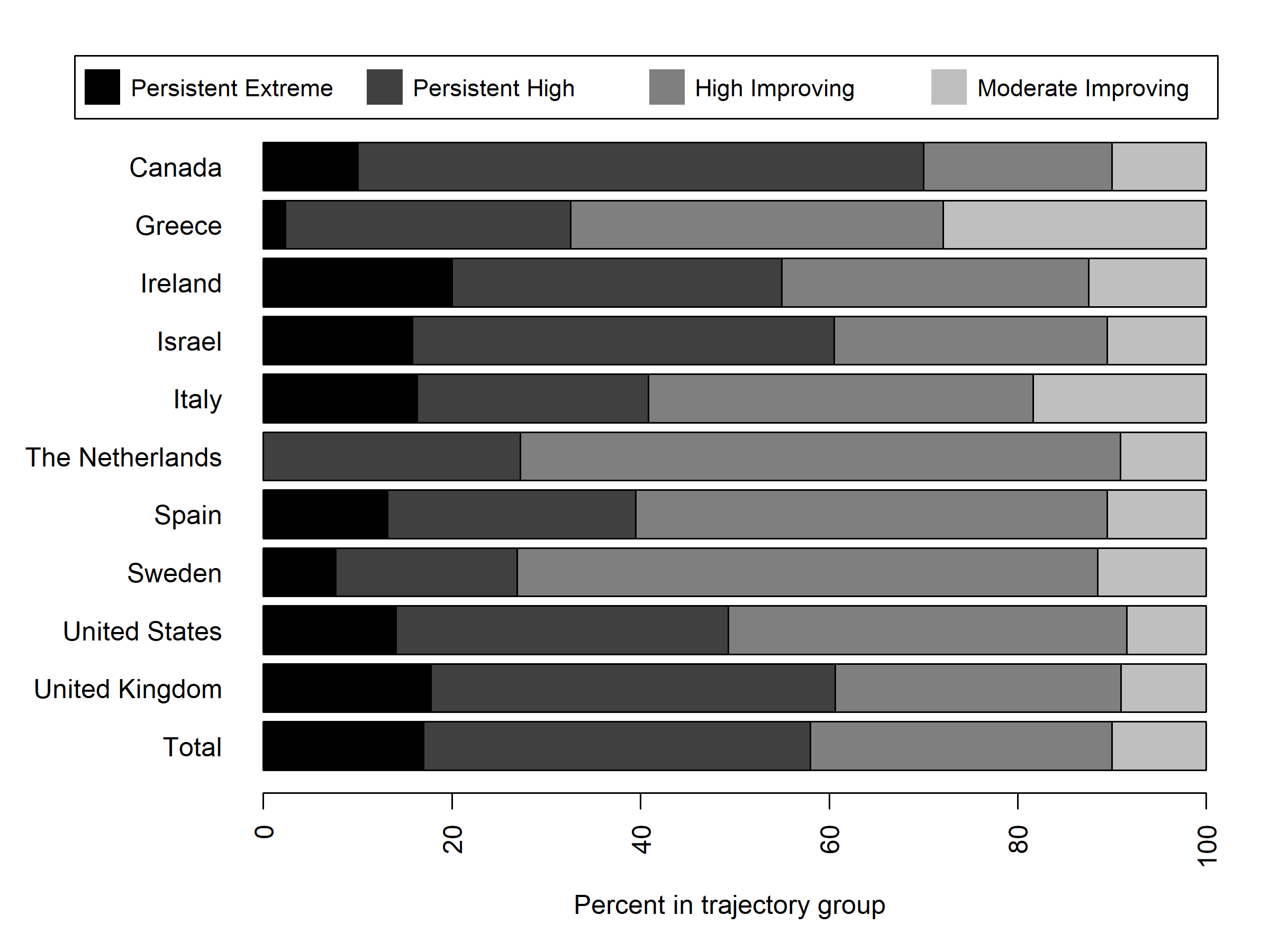


**Supplementary Figure S3.** Proportion of participants from each region of residence across the four trajectory clusters of COVID-19-related anxiety: a) Parents of children and young people, b) Adults.
